# Simulation Training in Medical Education—an Exploration Through Different Theoretical Lenses

**DOI:** 10.1007/s40670-019-00696-3

**Published:** 2019-02-06

**Authors:** Morkos Iskander

**Affiliations:** 1 Department of Educational Research, Faculty of Social Sciences, Lancaster University, Lancashire, UK; 2 Health Education North West, Liverpool, Merseyside UK

**Keywords:** Instructional design, Theoretical framework, Educational research, Research design

## Abstract

Different theoretical frameworks offer specific, but separate, understandings of the same phenomenon. With the increasing use of simulation for training and assessment in medical education, it is vital to consider how different frameworks grant various insights into the pedagogical value of simulation. In this article, the author evaluated three exemplar theoretical frameworks, cultural-historical activity theory, cognitive load theory, and grounded theory, considering their ontological and epistemological stances, their limitations, and their application to simulation training. The greater understanding offered by this article will inform research design and interpretation of results, enabling a more theoretically poised construction of pedagogical techniques.

## Background

Using a theoretical framework is a fundamental component of research in medical education. Choosing an appropriate theoretical framework can serve to articulate the research questions and the choice of methodology, as well acting as a lens to interpret findings [1–3]. Integration of theoretical approach to research findings serves as a platform to enable generalisation of the results [4, 5]. Each theoretical framework stems from a particular philosophical vantage point, and carries individual ontological and epistemic assumptions, and therefore dictates the questions that may be addressed and the expected outcomes [6–8]. Whether a particular framework is explicitly or implicitly acknowledged by study authors, it can be assumed to that one is imbedded and has a direct correlation with the research and its reported outcomes [9]. The advantage of choosing a theoretical framework early in the research process is the active appreciation of the limitations, insight into the research questions, and a more thorough awareness of the associated biases.

Simulation in medical education has been gaining increasing popularity over the previous three decades, with rising utilisation over time, indicating a realisation of the need for simulation to provide a simplified model of clinical practice to aid learning [10, 11]. Recently, the expectation that appropriate and comprehensive training will be undertaken before clinical practice, altered models of healthcare delivery, and coupled with a heightened focus on patient safety has led to an overall decrease in clinical exposure to direct clinical care, and thus fewer learning opportunities [12–14]. Simulation is seen as a solution to all these aspects through a focused curriculum, with a demonstrable impact on patient and behaviour [15, 16]. The educational value of simulation can be broadly divided into subcategories: the first of these is to provide a summative assessment in a standardised setting, with a view to ensure professional competence and the second setting is in the formative environment, providing an arena for deliberate practice and an opportunity to apply knowledge in a supportive environment, without exposing patients to risk.

In order to appreciate how the use of different theoretical frameworks may impact educational research in simulation training, I have considered below three major theoretical frameworks commonly deployed in simulation educational research. Each of the frameworks is summarised, the philosophical positioning is considered, and finally, its use for research in simulation is explored. In this section, I will present three theories that apply to educational research and their insights into simulation training, namely cognitive load theory (CLT), cultural-historical activity theory (CHAT), and grounded theory. The theories were chosen for comparison as they provide different perspectives on education and the learning process, as well as serving to inform different stages of research programs. Grounded theory may be applied when little or no theoretical understanding of the phenomenon being studied exists, CHAT affords a mechanism for understanding the integration of new learning into existing systems, and CLT can provide insight into the individual’s learning.

### Cognitive Load Theory

In this theory, it is argued that performance relies on the complex interplay between sensory inputs, long-term memory acting as a repository of acquired knowledge and skills, with working memory as the intermediate stage, acting to attribute meanings to the sensory information, and deposit new learned information into the long-term memory [17]. However, while both sensory and long-term memories are capable of dealing with large volumes of information, the capacity of working memory is comparatively very limited [18, 19]. Cognitive overload is presumed to occur when this capacity is exceeded, on occasions requiring the individual to coordinate a larger than possible number of elements to accomplish tasks successfully.

Dictating the direction of working memory is the individual’s metacognitive capacity, guiding working memory to relevant sensory information, as well as appropriate knowledge or schemas in the long-term memory, as well as managing different cognitive loads in order to avoid overload [20]. The level of metacognition functions by the monitoring and control of cognitive processes [21]. The value of appreciating the role of metacognition in managing the function of working memory has been highlighted in several studies, indicating that a higher level of performance may be attainable [22, 23].

At the core of this framework is a belief that there exists a set pattern that may be applied to human cognitive functions and be applied effectively across individuals and across fields. Furthermore, it is evident that learning, as contrasted to task completion, is accomplished by the development of generic schema that are applicable to a wider set of circumstances. In combination, these imply the existence of a definite reality from without the minds of the learners, with learning being the process of attempting to comprehend this reality more fully. However, as the schemas are liable to the biases inherent in the teacher and the student, it follows that the understanding of the reality may converge but is unlikely to coincide completely. It is therefore important to strive to minimise the effects of these inevitable biases in research. We can therefore conclude that cognitive load theory is a post-positivist philosophical approach [24].

From the ontological positions outlined above, the post-positivist stance stresses a pragmatist approach to the acquisition and accumulation of knowledge. The post-positivist paradigm embraces both qualitative and quantitative approaches to enquiry, holding the belief that a numerical approach is complemented by the qualitative approach [25, 26]. Knowledge, as opposed to reality, may therefore be considered to be subjective, and constructed from past and present experiences. The application to educational theory and instructional design is thus dependent on the assumption that an individual’s knowledge may be influenced by the learning process and guided to the ‘correct’ and objective truth. Following this line of argument forward, it becomes clear that the epistemological stance is therefore presumed rather than the absolute, with all hypotheses considered probabilistic and pending further review, with knowledge being relative to the individual.

Consequently, cognitive load theory is ideally suited to instructional design, providing a technique for developing teaching programs in simulation suited towards maximising the learning gain of trainees and students. Utilising a cognitive load theory affords a perspective that not only aids in the initial design of simulation training, but also in refining the simulation through evaluation and subsequent iterations. In analysing research results, cognitive load theory offers a reasoned approach to explaining performance during tasks. In their study of medical students, Young et al. [27] demonstrated that higher level performance in simulated clinical exercises is directly correlated to a lower related cognitive load. This understanding enables the development of strategies to reduce individual’s cognitive load, and therefore enhance performance.

### Cultural-Historical Activity Theory

The development of cultural-historical activity theory is rooted in the concept the interplay and relationship between the subjective experience and external world, as well as the dynamic relationship between them as they modulate one another. It describes how artefacts mediate the interaction between subjects and objects with learning and development occurring through a ‘zone of proximal development’, where skills are gradually improved and new ones acquired [28]. This is suggesting a gradual progression and increase in capability, with additional components of socio-cultural descriptors of activity systems. The interaction between the individual and the society, as mediated by the tools, results in the internalisation and externalisation processes. The concept of the zone of proximal development can also be applied to an activity system as a whole, as well as the wider community, describing how teams and groups may develop together [29].

The assumptions implicit in cultural-historical activity theory are that prior knowledge and the intrinsic organisational structure place limitations on individuals throughout the course of an activity. The interdependency between the individual and their environment therefore necessitates that they can only be studied when viewed as a ‘complete’ system; one cannot be meaningfully understood without the other. The implied limit is therefore that it can only be applied to the individual learner in a very limited sense. It can be used to understand how groups act but does not take account of the edges of the bell curve, the outliers, in a true sense.

Therefore, cultural-historical activity theory necessitates using an entire activity as the prime unit of analysis, a self-sufficient replicating entity [30]. The overall goal of the activity directs the actions of the individual components but lies outside the system itself. The invoking of an external reality dictating the goal of activity systems belies the philosophical origins of the theory with Karl Marx [31] and situates it between a positivist to post-positivist ontological position. The epistemological position here relies on empiricism and modification of activity systems through a process of ‘retooling’ to enable expansion into the zone of proximal development [32]. Empirical knowledge of the systems is mediated through the empirical measurements, and so accuracy is dependent on their precision. However, the systems may also be explored rationally, as well as experimentally. Deciphering this stance and understanding the implications leads us to place cultural-historical activity theory more towards the post-positivist spectrum.

The application of cultural-historical activity theory to simulation training is arguably most suited to developing teams and groups rather than individuals. In the application to medical practice and education, it has proven to be more pertinent to the system and organisational level analysis [33]. We can argue that it is due to the impact of this theory that leads to teams that work together to train together. Simulation research based on this theory is therefore suited to the mesoscale and macroscale, rather than that of the individual, and enables the incorporation of various factors to implement the desired change.

### Grounded Theory

This theory uses a social practice theoretical perspective, and thus the overarching common practices rather than individuals or institutional criteria were the analytical units. Grounded theory states that the actions and processes are the central component of human activity [34]. The core principles of grounded theory began as a method for drawing out the conceptual categories through the evaluation of social processes [35]. It is critical to acknowledge that there are three different active variants of grounded theory, ranging from a positivist, post-positivist, and finally through to a constructivist approach [36]. However, even the constructivist grounded theory retains links to the positivist origins, but may be referred to as weakly post-positivist, accepting a large degree of relativism [34, 37].

The starting point of grounded theory is that behavioural patterns in society in general follow set patterns, and this is particularly evident when considering definite groups [38]. Therefore, it aims at bringing these patterns to the surface, via examination and conceptualisation of participants’ actions, particularly as these inherent behavioural patterns directly lead on to subsequent behaviour. The value offered is compounded by the examination of the practices of a group, with conceptualisation dependent on being able to draw upon a large pool of primary sources [39]. This enables the grounded theory to surpass the individual’s sphere of practice and reaching the level of generalisability and abstraction. It has also been suggested when evidence implies that current theoretical understandings are deficient, a necessary emphasis is placed on obscure but meaningful experiences [40]. The choice of CGT for this study is therefore based on the combination of these advantages, coupled with the desire to base this template developed here on practice rather than theory alone.

The philosophical standpoint of grounded theory necessitates a belief in an objective, external reality, and it therefore sits with both the other theories explored here as on the positivist to post-positivist scale of ontology. Grounded theory’s approach does necessitate deviation from the ‘pure’ positivist philosophy far more than elsewhere. However, in contrast to cognitive load theory and cultural-historical activity theory, grounded theory is a posteriori, rather than the rationalist a priori approaches. A criticism of grounded theory has previously been just this point, citing the philosophical irreconcilability of a priori and a posteriori approaches. However, the constant comparison of grounded theory is dependent on both approaches, with an ongoing dialogue with theory, providing an effective mechanism for integration [41].

Grounded theory has therefore been used to develop understanding of different facets of simulation from an empirical basis. An example may be drawn from a cohort study, which used simulated scenarios with students that allow the scope for professional development within a safe environment [42]. This enables the composition of experimental observations into a coherent theoretical understanding of the simulation, and therefore may lead on to a more refined iteration of the simulation. This process may enable the fine tuning of simulations to promote specific aspects for more accelerated and focussed learning.

## Conclusions

The choice of which theoretical framework used in a research project is dependent on several factors. Initially, the ontological and epistemological stances need to coincide with those of the research team, or at the very least avoid outright contradiction with them [43]. Having decided on the framework and the problem to be researched, the structure of the research program is then apparent, as these serve as the blueprint that dictates the steps to follow [1]. As the results are also viewed through the same lens, it is therefore imperative not to underestimate the care that must be taken in making an appropriate theoretical framework. Different theoretical frameworks will shed a different light on a topic, as they view and understand it from differing perspectives. Table 1 summarises the three theories expounded in this article. Alignment between the research questions and the theoretical framework will ensure that the results from specific projects may be expanded and generalised.

**Table 1:** Aspects of the three theoretical frameworks

	CLT	CHAT	Grounded theory
Ontology	Post-positivist	Post-positivist	Post-positivist
Epistemology	A priori*/theoretical*	A posteriori*/empirical*	A posteriori*/empirical*
Level	Micro	Meso/macro	Micro/meso
Goal	Optimise learning through minimising non-essential aspects	Understand the deficient knowledge and skill in a system, facilitate development	Develop a theoretical understanding of simulation training through extrapolation
Purpose	Predict and control learning processes	Establish connections between different aspects	Stimulate developments

It may be that *compatible* theoretical frameworks may be utilised, either sequentially or in parallel, to examine the same phenomenon from a different angle, with each lens offering particular insights. This necessitates that researchers become intimately familiar with the chosen framework, its philosophical stances, and limitations.
